# Proceedings of the Hahnemannian Association of Pennsylvania

**Published:** 1888-02

**Authors:** Wm. Jefferson Guernsey

**Affiliations:** Secretary


### PROCEEDINGS OF THE HAHNEMANNIAN ASSOCIATION OF PENNSYLVANIA.

The Hahnemannian Association of Penna. met in the Continental Hotel, Philadelphia, January 10th.

Dr. Mahlon Preston, of Norristown, presiding.

Present, four active members, four associates, three honorary members, and two visitors.

Dr. C. F. Tegtmier, of Conshohocken, was elected to associate membership.

Dr. Wm. H. A. Fitz, of Philadelphia, read a paper on *Colchicum autumnale.*

Discussion:

Dr. C. Carleton Smith—We often have to differentiate between this drug and *Mercury*, but the shreddy stool and sour sweat of the former helps us out.

*Colch.* is the base of all quack rheumatic remedies. I had a patient once die after taking one of these, and she complained fearfully of "pains in the small joints." This is a marked symptom of the drug, and shows a night aggravation, the pains becoming worse in the evening. *Rhus* has an evening aggravation which lasts till three o'clock. *Merc.* is similar, but sweats more.

Dr. Mahlon Preston—*Colch.* has profuse sweat also.

Dr. Smith—The nausea from smell of food cooking is an important symptom, but must be stated by the patient voluntarily to be of value, and not elicited by inquiry; it must be marked.

Dr. Clarence Willard Butler (Montclair, N. J.)—What are the pains in small joints?

Dr. Smith—Tearing and laming.

Dr. Butler—I find a stiffness marked, especially if with the sour sweat.

Dr. Preston—What is your experience with the eye symptoms?

Dr. Smith—Like *Merc.*, worse in the open air.

Dr. Preston—I once made a nice cure with *Colch.* of an ulcer of the cornea, which was surrounded by a high growth like the mercurial ulcer. It was in a child of four years, and three or four doses of the 200th cured the trouble in two weeks.

Dr. J. H. Hamar—What indication had you for *Colch.?*

Dr. Preston—The position of the ulcer. You will find it mentioned in the *Symptomen Codex.*

Dr. John V. Allen—I once cured a case of neuralgia of the stomach with that sensation of coldness in the stomach.

Dr. Wm. Jefferson Guernsey—*Ambra grisea* has that coldness in stomach also. I had a patient who had suffered for thirteen or fourteen years from this symptom, winter and summer, having been exposed in a sleigh ride. He had used all kinds of pads and warmers without relief, and was better in twenty-four hours after taking the *Ambra.**

Dr. Allen—I had a patient with coldness, as if freezing in the stomach, to whom I gave *Phos.* for some chest symptoms, and on looking up that drug found it also had the coldness. *Phos.* cured *all* his symptoms.

Dr. Butler—Some years ago a young man came to my office with clap. It was about meal-time, when the odor from the kitchen was quite noticeable. He was taken with an attack of excessive nausea from it, and on this indication I gave him *Colch.*, which cured his nausea and clap, too.

Dr. R. B. Johnstone, of Germantown, next read note 126 from the appendix to the *Organon.*

Disscussion ;

Dr. Allen—I can't see the difference between a single globule and a dozen as a dose.

Dr. Johnstone—I find, sometimes, that medicines do not always act just as I had expected, and have thought that it might be because in replenishing the vial over and over with alcohol I was producing a potency of alcohol as well as of the drug.

Dr. Guernsey—You can't potentize a menstruum with itself.†

Dr. Samuel Long (New Brunswick, N. J.)—What quantity of globules would you use as a dose?

---

* This man has since had slight recurrences of the trouble, always helped *at once* by *Ambra.*—G.

† It is worth thinking of, however, that in drugs, run up to the third or fourth by trituration with sugar of milk and *afterwards* by alcohol, that there may be a potentization of *Saccharum lactis* with the drug.—G.

Dr. Smith—I use a few of the smallest size, No. 5.

Dr. Long—Dr. Constantine Lippe never had a liquid in his house, but always added to his medicines by dry succussion.

Dr. Smith—I think we can give an overdose. Dr. Dunham once said one globule was as good as a thousand, and while it does not prove the opposite, I think he had that in his mind.

Dr. Guernsey—I will not use sugar of milk on account of its taste. It resembles Magnesia so strongly that any patient under treatment for constipation would swear that he was taking that drug.

A letter was then read from Dr. Geo. H. Clark, asking for advice in a difficult case.

Dr. Guernsey read a brief paper on "The Quinine Curse."

Dr. Johnstone—What remedy has the symptom—imagines that some one has poured a poisonous substance on the skin?

Dr. Guernsey—*Hyos.* has fear of being poisoned, which is similar.

Appointments for next meeting:

Materia Medica, Dr. Horace Still, of Norristown, who will present a paper on *Ambra grisea.*

*Organon* dissertation, Dr. C. Carleton Smith, Philadelphia.

Original paper, Dr. C. H. Lawson, of Wilmington.

WM. JEFFERSON GUERNSEY, *Secretary.*

